# Clinical Application of Botulinum Neurotoxin in Lower-Urinary-Tract Diseases and Dysfunctions: Where Are We Now and What More Can We Do?

**DOI:** 10.3390/toxins14070498

**Published:** 2022-07-18

**Authors:** Hann-Chorng Kuo

**Affiliations:** Department of Urology, Hualien Tzu Chi Hospital, Buddhist Tzu Chi Medical Foundation, Tzu Chi University, Hualien 97004, Taiwan; hck@tzuchi.com.tw; Tel.: +886-3-8561825 (ext. 2117); Fax: +886-3-8560794

**Keywords:** adverse events, lower-urinary-tract dysfunction, therapeutic efficacy, urinary incontinence, voiding dysfunction

## Abstract

Botulinum toxin A (Botox) had been considered a promising drug that has an effect on functional disorders of the lower urinary tract. Because Botox exhibits anti-inflammatory and antispasmodic effects, Botox injection into the bladder can decrease detrusor contractility, reduce bladder hypersensitivity, and eliminate painful sensations. Injecting Botox into the bladder outlet can relax the hyperactivity of the bladder neck, and of the urethral smooth and striated muscles. Based on these therapeutic effects, Botox has been widely applied to treat lower-urinary-tract dysfunctions (LUTDs) such as overactive bladder and neurogenic detrusor overactivity. However, this treatment has not been licensed for use in other LUTDs such as interstitial cystitis, voiding dysfunction due to benign prostatic hyperplasia in men, and dysfunctional voiding in women. Botox has also not been approved for the treatment of children with overactive bladder and dysfunctional voiding; in patients with spinal cord injuries with detrusor sphincter dyssynergia and autonomic dysreflexia; or for poorly relaxed external sphincter in non-neurogenic patients. This article reviews the current knowledge regarding Botox treatment for LUTDs and discusses the potential clinical applications of Botox, as well as work that can be conducted in the future.

## 1. Introduction

In recent decades, botulinum toxin A (Botox) has been widely used for the treatment of several different lower-urinary-tract dysfunctions (LUTDs), including overactive bladder (OAB), neurogenic detrusor overactivity (NDO), interstitial cystitis/bladder pain syndrome (IC/BPS), pediatric urinary incontinence and voiding dysfunction, voiding dysfunction due to benign prostatic hyperplasia (BPH) in men, dysfunctional voiding (DV) in women, detrusor sphincter dyssynergia (DSD) in patients with spinal-cord injury (SCI), and poorly relaxed external sphincter (PRES) in non-neurogenic patients. Despite the fact that Botox injection has only been licensed to treat OAB and NDO refractory to conventional treatment, the clinical applications of Botox on other LUTDs have been enthusiastically tried [1]. However, because some clinical experiences of adverse events have limited its widespread application, Botox injection treatment has not popularly used for LUTDs other than OAB and NDO. Nevertheless, evidence has shown that Botox has distinguished advantages related to functional alteration, chronic inflammation, and sensory disorders in some LUTDs that are difficult to treat with oral pharmacologic medications. Other than OAB and NDO, several other LUTDs can benefit from intravesical Botox injection, intravesical instillation, or urethral Botox injection. (Table 1) This article focuses on the off-label and novel applications of Botox on LUTDs. The content of this article may provide updated knowledge and information regarding the current situation and where Botox may be further applied in functional urology and LUTDs.

## 2. Clinical Application of Botox on OAB: Efficacy, Adherence, Adverse Events, and Novel Treatment without Injection

Botox treatment for OAB and NDO is becoming increasingly recognized as an effective therapeutic option for patients who are refractory to or cannot tolerate anticholinergic agents. The results from open-label studies have suggested that this therapy is effective in both neurogenic and idiopathic detrusor overactivity (DO) [2]. However, undesired adverse events and the need for repeat injections remain obstacles to the popularity of this treatment.

Botulinum toxin is a neurotoxin produced by *Clostridium botulinum* that inhibits signal transmission at the neuromuscular and neuroglandular junctions [3]. The most popular current clinical use of Botulinum toxin is onabotulinumtoxinA (Botox^®^, Allergan, Irvine, CA, USA). Botox has received regulatory approval for LUTDs, including OAB and NDO. The approved dose of Botox for NDO due to SCI or multiple sclerosis (MS) is 200 U by 1 mL for each injection into the detrusor at 30 sites on the bladder wall. For Botox treatment of OAB, the dose is 100 U by 0.5 mL for each injection into 20 sites on the bladder wall [4]. Botox has both a motor and sensory effect on the lower urinary tract; therefore, the therapeutic effects include not only a decrease in striated or smooth muscle contractility, but also effects on sensory dysfunction, including frequency urgency sensation and bladder pain [5,6].

Following the initial clinical trials of Botox injection for OAB, the recommended optimal dose was set at 100 U injected into 20 sites of the bladder wall [7,8]. Adverse events of this treatment include difficult urination, urinary-tract infection (UTI), and large postvoid residual (PVR) volume requiring clean intermittent catheterization [9]. For patients who are frail and old with a PVR > 100 mL, injecting at the bladder base and trigone is safer to avoid acute urinary retention and subsequent UTI [10]. Phase 3 clinical trials and pooled analysis have demonstrated that Botox injection is superior to placebo and that patients may have fewer episodes of urgency and urgency urinary incontinence (UUI) as well as experiencing an improvement in quality of life [11,12].

### 2.1. Adherence of Botox Treatment in OAB and Causes of Discontinuation

According to the guidelines of the American Urological Association and recommendations of the European Urological Association, Botox intravesical injection is the third-line treatment for OAB [13,14]. Repeated Botox injections have been reported to have a similar effect, and the interinjection interval has been reported to remain unchanged for up to five injections [15]. However, increasing age, a Botox dose of 200 U, higher body-mass index, and baseline UUI episodes were found to be associated with a shorter time to UUI recurrence after Botox injection [16]. In patients who have diminished Botox effect on OAB symptoms, adding mirabegron could increase the therapeutic effects, mainly on OAB symptoms and the Global Response Assessment scale [17].

A long-term follow-up study revealed a low rate of persistence of another brand of botulinum toxin A, abobotulinumtoxinA, as an injection for OAB. A total of 59.3% of patients were successfully treated with the first injection. The median number of injections per patient was only two, and the median reinjection interval was 10.7 months. The estimated 5-year discontinuation-free survival rate was 23.4%. The main cause of discontinuation was primary failure in 35.5% of patients, 23.7% of patients had persistent symptom improvement, and 20.3% stopped the injections because of tolerability issues [18]. In women with OAB, a significant reduction in the DO rate and an increase in the median maximum cystometric capacity were noted after Botox injection for idiopathic DO. The maximum flow rate (Qmax), detrusor pressure (Pdet), and PVR all showed no significant change, and no patient required catheterization after Botox injection [19].

### 2.2. Comparison of Repeat Botox Injections and Percutaneous Tibial Nerve Stimulation and Sacral-Nerve Neuromodulation

In addition to Botox injections, percutaneous tibial nerve stimulation (PTNS) and sacral-nerve neuromodulation (SNM) are two options for the treatment of refractory OAB. Because Botox injection requires local or general anesthesia, and repeated injections are necessary to maintain the therapeutic effect, patients might consider switching to another procedure for better convenience. A recent study revealed that PTNS and Botox resulted in a similar improvement in OAB symptom scores; however, Botox resulted in significantly greater improvement in urgency and UUI episodes than PTNS [20]. Overall, Botox, PTNS, and SNM were more efficacious than the placebo. However, the greatest reduction in UUI episodes and voiding frequency was observed with SNM. Botox resulted in a higher complication rate, including UTI and urine retention, as compared with PTNS [21].

Although SNM is an invasive procedure and requires a two-stage implantation, the therapeutic effectiveness is as high as 69%, and the battery lasts for up to 15 years [22]. SNM has become a well-accepted procedure for refractory OAB, especially in women. However, the high cost of SNM limits its wide application in treating UUI as compared with 200 U of Botox [23]. In patients who failed previous Botox treatment and in Botox-naïve patients, the therapeutic efficacy was shown to be similar between the Botox and SNM groups and similar between patients with a previously different volume of Botox injection [24]. Although the risks of UTI and urinary retention are higher in patients treated with Botox, the risk of revision or removal of the battery and implant also requires caution [25].

### 2.3. Potential Vehicle to Carry Botox across the Urothelium without Injection

Liposome-encapsulated Botox intravesical instillation had been demonstrated as effective in decreasing OAB symptoms without adverse events such as a PVR increase or the risk of UTI [26,27]. An immunohistochemistry study showed that Botox injection can effectively cleave the SNAP-25 protein, whereas liposome-encapsulated Botox can decrease urothelial P2 × 3 expression but does not cleave SNAP-25 [28]. The same formulation of liposome-encapsulated Botox had been applied in the treatment of patients with IC/BPS, and the results showed a positive effect on the decrease in IC symptoms, although the improvement was not superior to the placebo arm [29]. Nevertheless, these pilot studies demonstrated that, using liposomes encapsulation, the Botox protein can be delivered across the phospholipid layer of the cell membrane and act on the sensory receptors of the urothelial cells. However, the depth of Botox penetration might be too superficial to achieve a longer therapeutic effect. Thus, additional investigation of the treatment frequency and dosage of Botox are necessary [30].

LESW was shown to be effective for temporarily increasing tissue permeability and the intravesical delivery of Botox for the treatment of OAB in animal studies and in a human clinical trial [31]. Our preliminary study using LESW and intravesical BoNT-A instillation every week in OAB patients also demonstrated an improvement in the Global Response Assessment without any adverse events [32]. A prior immunohistochemistry study revealed the presence of cleaved SNAP-25 protein in the IC bladder suburothelium, suggesting that Botox molecules could be carried across the urothelial cell membrane with the assistance of LESW. These results provide evidence of the efficacy and safety of this novel treatment using LESW plus Botox instillation without anesthesia or bladder injection [32].

### 2.4. Perspectives of Researchers on Botox Injection Related to UTI, Difficult Urination, and Adverse Events after Treatment

The most common and frustrating adverse events after Botox injection for OAB are UTI and dysuria [33]. In patients with OAB, previous UTI is the strongest predictor of UTI after Botox injection. Men have 2.4 times higher odds of incomplete emptying than women, and 17% of men and 23.5% of women experience more than one episode of UTI in the first month following injection [34]. Although researchers have reported that aging is associated with a higher rate of large PVR and lower long-term success [35], the age-related outcomes of Botox for the treatment of OAB are significantly understudied [36]. Male gender, baseline large PVR (≥100 mL), comorbidity, and a Botox dose of >100 U are risk factors for increasing the incidence of adverse events after intravesical Botox injection for idiopathic detrusor overactivity [37]. Factors that can predict poor response and higher risk of UTI to Botox injection for OAB include female gender, retained prostate in men, and clean intermittent self-catheterization [38]. A recent meta-analysis also revealed a positive effect of Botox treatment on sexual function in patients with OAB. Significant improvement was observed in desire, arousal, lubrication, orgasm, and satisfaction after Botox injection; however, there was no improvement in pain [39].

Based on the results of previous studies, several topics of research remain to be investigated: (1) Can a small dose of Botox injection and combined medications (mirabegron or antimuscarinics) be used to treat patients with OAB to avoid undesired UTI and urinary retention? (2) Can the dose of Botox be flexibly adjusted to fit the needs of patients with different severities of OAB and neurologic lesions? (3) Can we use LESW on the bladder plus Botox instillation to treat patients with OAB or hypersensitive bladder without intravesical injection under anesthesia?

## 3. Clinical Application of Botox on NDO: Efficacy, Adherence, Adverse Events, and Conversion of Treatment

OnabotulinumtoxinA was first injected into the urethral sphincter to treat patients with SCI with DSD who did not desire surgery or were unable to perform self-catheterization [40]. Later clinical trials confirmed the therapeutic effectiveness of 200 U or 300 U of Botox detrusor injections on NDO due to SCI and MS [41,42]. In patients with Parkinson’s disease and SCI, Botox detrusor injection can modulate bladder afferent activity, which explains why Botox can improve DO [43]. The therapeutic duration is about 6 to 9 months, and the significant reduction in detrusor pressure, increase in bladder capacity, improvement in hydronephrosis, and reduction in UTI episodes are remarkable after Botox injection [42,44]. After several repeated detrusor Botox injections, the therapeutic efficacy remains the same [45]. Currently, Botox detrusor injections are widely applied in the treatment of patients with chronic SCI or MS refractory to antimuscarinic therapy for NDO and UUI [46,47,48].

### 3.1. Therapeutic Efficacy, Adverse Events, and Tolerability of Botox Injection for NDO

In a post-market survey, intradetrusor Botox injections were reported to be safe and to be able to improve subjective measures related to NDO [49]. A meta-analysis also revealed that Botox can result in a significant reduction in UUI frequency and improvement in urodynamic parameters in patients with NDO at 6 weeks after treatment. No statistical or clinical difference in efficacy has been reported between 300 U and 200 U dosages of Botox [50]. Patients treated with 300 U of Botox had similar therapeutic efficacy after 200 U of Botox treatment. The number of episodes of urinary incontinence and daily pad use were similar between the two dosage phases [51]. However, quality-of-life measures were significantly improved, and an improvement in end-filling pressure and bladder compliance was also reported [52]. Although Botox detrusor injections are effective and tolerable for patients with NDO, adverse events related to Botox injection, including hematuria, UTI, urinary retention, urinary bladder hemorrhage, autonomic dysreflexia (AD), and epididymitis, warrant caution [53]. The results of a long-term follow-up study showed that only 50% of patients with SCI continuously received intradetrusor Botox injections for NDO after 10 years [54]. In patients with NDO, repeat Botox injections allow sustained improvements in UUI, with an acceptable rate of adverse events [55]. The efficacy of repeat detrusor Botox injections included significant improvement in urinary symptoms and bladder compliance in 52% of patients, and 31% of patients had an objective improvement in bladder compliance [56].

### 3.2. Switching from Botox to Augmentation Enterocystoplasty

After the initial Botox injections for NDO, some patients might not experience persistent improvement in symptoms, and some might prefer to have definite treatment without repeated injections. Augmentation enterocystoplasty (AE) is one option for patients with SCI and NDO and urinary incontinence [57]. Patients receiving AE had a statistically significant increase in bladder capacity and a decrease in detrusor pressure during voiding. Patients with SCI receiving Botox injections but who experience few improvements in their urodynamic parameters should consider switching from repeat Botox injections to AE to achieve better storage function and functional bladder capacity [58]. In patients who still have symptoms of NDO or AD after AE, the injection of Botox into the native bladder was effective in 58% of patients in increasing bladder capacity and decreasing detrusor pressure [59].

### 3.3. Causes of Discontinued Botox Injection in OAB and NDO

Although Botox injections are effective for treating urinary incontinence of OAB and NDO, more than half of patients with NDO discontinue Botox injections within the first 10 years after their first treatment [60]. Patients with spina bifida have a higher discontinuation rate. The most common cause of discontinuation is treatment failure (43.7%). In a long-term follow-up study of the satisfaction rate of patients with SCI and NDO who received detrusor Botox injections, only 48.4% of patients continued Botox injections over 7 years [61]. The presence of high detrusor pressure and higher-grade bladder outlet resistance are predictive of a decrease in incontinence. In total, 69.1% of patients expressed satisfaction with their current status.

## 4. Clinical Application of Botox on Interstitial Cystitis/Bladder Pain Syndrome: Efficacy, Adverse Events, and Perspectives

### 4.1. Current Strategy of Using Botox Injections on IC/BPS

IC/BPS has been classified into classic ulcer and non-ulcer types based on cystoscopic findings [30]. Although researchers have proposed many pathogeneses of IC/BPS, the actual etiology remains unclear. Possible etiologies include (1) the post-infection autoimmune process; (2) mast-cell activation induced by inflammation, toxins, or stress; (3) urothelial dysfunction and increased permeability of the urothelium; and (4) neurogenic inflammation resulting in increased urothelial permeability, mast-cell activation, the up-regulation of sensory fibers, the release of inflammatory neuropeptide, and bladder pain [62]. In addition, psychosomatic dysfunction has also been reported to be involved in the pathophysiology of IC/BPS, especially in the pain phenotype [63,64]. Conventional therapies include oral pentosan polysulfate [65], intravesical heparin instillation [66], intravesical hyaluronic acid instillation [67], and oral medications that target suburothelial inflammation. Although IC/BPS has not received regulatory approval for Botox use, current evidence supports that Botox injection can improve symptoms and bladder pain for IC/BPS as compared with a placebo [68].

Intravesical onabotulinumtoxinA injections might not only reduce bladder sensitivity in patients with IC/BPS but also induce desensitization in the central nervous system by affecting the overexpression of activated proteins in the dorsal horn ganglia [69]. The therapeutic effects of Botox on IC/BPS were confirmed by several clinical trials showing that Botox could effectively reduce bladder pain and urinary frequency and improve psychosocial functioning [70,71,72]. As compared with cystoscopic hydrodistention, intravesical Botox injection plus hydrodistention can improve IC symptom scores, reduce bladder pain, and increase functional bladder capacity [73]. In the first prospective, multicenter, randomized, double-blind, placebo-controlled clinical trial, we demonstrated a significantly greater reduction in pain in the Botox group compared with the normal saline group (−2.6 ± 2.8 vs. −0.9 ± 2.2, *p* = 0.021) at week 8. We also found that the cystometric bladder capacity was increased significantly in the Botox group. The overall success rates were 63% in the Botox group compared with 15% in the control group (*p* = 0.028), with a similar rate of adverse events between groups [74].

The injection of Botox into the pelvic floor muscles of women has also been shown to improve chronic pelvic pain syndrome; however, this treatment may worsen preexisting pelvic floor conditions such as constipation, stress urinary incontinence, and fecal incontinence [75,76].

### 4.2. Predictive Factors for a Successful or Failed Treatment Outcome

In patients with non-ulcer IC/BPS, intravesical Botox injections can effectively improve symptoms and reduce bladder pain, but these injections do not benefit patients with ulcer IC/BPS [77]. As compared with a single injection, repeat intravesical Botox injections were associated with a significantly higher success rate in a long-term follow-up. However, the incidence of adverse events did not increase with a higher number of Botox injections. A higher pretreatment interstitial cystitis symptom index and interstitial cystitis problem index score were predictive of a successful response to repeated intravesical Botox injections [78]. Injecting Botox into the bladder body and trigone did not result in a difference in treatment outcomes or rates of adverse events [79].

In a recently published analysis of factors predictive of successful Botox treatment outcomes for IC/BPS, patients with a maximal bladder capacity under hydrodistention of >760 mL had a satisfactory treatment outcome [80]. The improvement in bladder pain was remarkable in patients with a satisfactory treatment outcome. A maximal bladder capacity of ≥760 mL is a predictive factor for satisfactory treatment outcome, whereas glomerulation grade and urodynamic parameters do not have a predictive value for the IC/BPS treatment outcome. Only 10% of patients who were treated with Botox injection complained of difficulty in urination after treatment.

### 4.3. Comparison of Effects between Different Botox Injection Sites and between Botox and Sacral Neuromodulation in Treatment of IC/BPS

There has been debate regarding the effectiveness of Botox on IC/BPS between different sites of injection. The trigone has been considered to be rich in unmyelinated nociceptive C-fibers, and it may be a potential target for chemodenervation in patients with IC/BPS [81]. Giannantoni et al. injected Botox at the trigone and lateral bladder wall and reported symptomatic improvement in 86.6% of patients [70]. Pinto et al. injected 100 U of Botox into 10 trigonal sites and found an increase in bladder capacity and a transient reduction in urinary nerve growth factor and brain-derived neurotrophic factor [82]. However, in our study, after intravesical Botox injection in the bladder body or trigone, we did not find any significant difference in improvement in IC symptoms or urodynamic parameters [79]. Evans et al. compared the treatment outcomes between patients with IC/BPS who were randomly assigned to a trigone-including or trigone-sparing Botox injection template. They found no significant difference between groups or in the post-treatment complication profiles [83].

### 4.4. Perspectives of Future Research on Botox Treatment IC/BPS: Using Instillation with the Aid of LESW or Liposome but Not Injection

Although intravesical Botox injections of IC/BPS have been shown to be significantly superior to intravesical Botox instillation, the adverse event of difficult urination after Botox injection remains a problem to be solved [84]. The delivery of Botox via liposome encapsulation and gelation hydrogel intravesical instillation provided a potentially less invasive and more convenient form of application for patients with IC/BPS [85]. However, a pilot study showed only a short-term effect and limited improvement in IC symptoms in liposome-encapsulated Botox treatment [29]. Using LESW to increase urothelial permeability and facilitate the penetration of Botox intravesical instillation showed an early promising effect and the presence of cleaved SNAP-25 protein in the suburothelium [28]. These results provide evidence for the future treatment and safety of this novel treatment modality for patients with IC/BPS using LESW plus Botox instillation without bladder injection under anesthesia injection [32].

## 5. Clinical Applications of Botox on Pediatric OAB and DV: Efficacy, Adherence, and Adverse Events

### 5.1. Advantages of Botox Injection for Pediatric Refractory OAB

Although Botox is not licensed for use in children with neurogenic or non-neurogenic LUTD, this treatment has been widely applied to treat pediatric OAB and DV refractory to conventional therapy. At a dose of 5 U to 10 U/Kg detrusor injection in patients with myelomeningocele and NDO, an increase in bladder capacity, and a decrease in detrusor pressure were noted [86,87]. Vesicoureteral reflux resolved in 73% of patients after detrusor Botox injections [88]. An additional urethral sphincter Botox injection at a dose of 2 U/Kg could decrease PVR in children with DSD [89]. Pediatric patients with non-neurogenic OAB can also benefit from detrusor Botox injection for the reduction of urinary incontinence [90,91,92].

In children with urinary incontinence due to NDO or OAB, Botox detrusor injection results in a significant improvement and is well tolerated [93]. A high rate of urinary continence can be achieved, with improvements in urodynamic parameters, including a reduction in detrusor pressure to <40 cmH_2_O and an increase in compliance to >20 mL/cmH_2_O, without major adverse events. In children with NDO, a dose of 200 U of Botox was well tolerated and showed greater efficacy in bladder pressure reduction and bladder capacity increase [94]. Children with a high detrusor pressure and low bladder compliance had a significantly greater improvement in compliance after detrusor Botox injections [95]. It was reported that the therapeutic effects persisted in the first 6 months after Botox injection in children with severe OAB symptoms, urodynamic-confirmed DO, and reduced bladder volume [96]. When children are treated with Botox injections for OAB, mirabegron and anticholinergics may be used as an exit strategy for recurrent OAB symptoms after the Botox effect declines [97].

### 5.2. Do Children with OAB Need Continuous Repeated Botox Injections? Will OAB Resolve with Aging?

For children with neurogenic or non-neurogenic DO, repeat intravesical Botox injections are required to maintain therapeutic efficacy. In children with NDO, Botox may provide long-term urinary continence and upper urinary tract protection, and the results remain effective after up to 11 injections [98]. However, in children with DV or DSD, the response to Botox was less predictable, with <50% of patients experiencing symptom resolution [98]. In children with myelomeningocele, repeated Botox injections were found to be safe and effective for keeping the bladder and upper urinary tract in a stable condition [99]. A systematic review and meta-analysis also confirmed that repeated Botox injections provided sustainable improvement in children with NDO and an acceptable rate of adverse events [55]. Nevertheless, the failure rates were reported to be 12.6% after 3 years, 22.2% after 5 years, and 28.9% after 7 years of follow-up with a withdrawal rate of 11.3%. Patients with severe NDO at baseline might experience a less favorable treatment outcome [100]. For this reason, a prior study reported that, based on long-term follow-up, a bladder procedure was still required in 35.3% of children with NDO and a severely contracted bladder that could not be recovered to a stable condition after repeated Botox detrusor injections [101].

### 5.3. Therapeutic Efficacy of Urethral Sphincter Botox Injections for Children with Non-Neurogenic DV

DV is highly prevalent in pediatric patients with NDO or non-neurogenic OAB [102]. Urethral sphincter Botox injection at a dose of 50 to 100 U can normalize the voiding curve and decrease PVR in children with non-neurogenic DV [103,104]. In children with DV, a higher dose of Botox may increase the therapeutic efficacy without increasing morbidity. Urethral sphincter Botox injection appears to be effective and safe in treating voiding dysfunction in children with DV or DSD [105].

## 6. Clinical Applications of Botox on Neurogenic and Non-Neurogenic Voiding Dysfunction: Efficacy and Perspectives

### 6.1. Current Treatment Outcome of Urethral Sphincter Botox Injection on DSD

Botox urethral-sphincter injection was first applied in patients with DSD due to SCI and MS [106]. Despite the lack of a phase 3 clinical trial and of licensed approval for clinical use, Botox has been widely applied for treating voiding dysfunction due to neurogenic or non-neurogenic voiding dysfunctions [107,108]. After the urethral sphincter Botox injection, patients with DSD or DV showed an improvement in voiding efficiency and a reduction in UTI episodes [109,110]. The dose of Botox for urethral-sphincter injections ranged from 100 U to 200 U for voiding dysfunction in patients with MS, cerebrovascular accident, or SCI [107,111]. After a urethral injection of 100 U of Botox, the indwelling catheter or clean intermittent catheterization can be discontinued in most patients [107,112]. In male patients with SCI with NDO and DSD who have received concomitant detrusor and urethral sphincter Botox injection, a significant reduction in detrusor pressure and maximal urethral closure pressure and an increase in maximal cystometric bladder capacity at 3 months after treatment were noted [113]. A better improvement rate was observed in patients with cervical SCI, the presence of NDO and DSD, partial hand function, and incomplete SCI. However, only 35.6% of patients continually received urethral sphincter Botox injections, and >60% of patients converted their treatment to another bladder outlet surgery to facilitate spontaneous voiding [57]. A meta-analysis showed that Botox was effective in 60–78% of patients with DSD for reducing PVR and lowering detrusor pressure and detrusor leak-point pressure after treatment. To maintain the therapeutic effect, reinjection is required after 4–9 months, without significant adverse events [114].

### 6.2. Current Treatment Outcome of Urethral Sphincter Botox Injection on Non-Neurogenic Voiding Dysfunction

In neurologically normal women, DV is characterized by an intermittent and/or fluctuating flow rate due to nonrelaxing or involuntary intermittent contractions of the periurethral striated or levator muscles during voiding [115]. Non-neurogenic DV may occur due to an enhanced guarding reflex against uninhibited detrusor contractions during the storage phase [116,117]. Treatment modalities for female DV include biofeedback pelvic-floor muscle training, medications such as alpha-blockers and skeletal muscle relaxants to decrease urethral resistance, antimuscarinic drugs for DO, and urethral Botox injection [118]. However, a urethral sphincter Botox injection can relieve voiding dysfunction in only about 67.9% of patients with DV. Patients with a tight bladder neck or with detrusor underactivity and low abdominal pressure to void might have a poor therapeutic result [119]. Multivariate analysis also revealed that narrowing of the bladder neck and history of catheterization were predictive factors for a negative outcome [120]. Another study reported a successful outcome in 59.4% of patients. There was no difference in treatment outcome between the different genders, voiding dysfunction subtype, bladder dysfunction, or sphincter dysfunction subtypes. In patients with DV, a significantly higher detrusor pressure might predict a successful treatment outcome of urethral sphincter Botox injection [121].

### 6.3. Perspectives of Urethral Sphincter Botox Injections for Patients with DSD or DV

Although urethral sphincter Botox injections comprise a safe and effective treatment for patients with urethral sphincter hyperactivity, the success rate has not been satisfactorily high. Currently, only 60% of patients with neurogenic DSD or non-neurogenic DV have benefited from this treatment [57,119]. No study has investigated whether an increase in Botox dose might result in a higher treatment success rate. In addition, because the pathophysiology of DV remains unclear, repeat urethral sphincter Botox injections could eliminate chronic inflammation in the central nervous system via the mechanism of afferent desensitization. Based on previous clinical trials of Botox injection at the trigone for eliminating bladder pain and sensory frequency in patients with IC/BPS [70,82], we might hypothesize that in patients with DV, injecting Botox into the trigone and bladder neck results in better therapeutic success than injecting into the urethral sphincter. Therefore, the following topics are of interest and could be investigated in the future: (1) whether the dose of Botox (100 U) and injection interval (once every 6 months) are enough to treat voiding dysfunction; (2) the adherence of urethral Botox injection for voiding dysfunction; and (3) whether voiding dysfunction improves with Botox injections into the trigone and bladder base, rather than into to the urethral sphincter.

## 7. Clinical Applications of Botox on Male LUTS and BPH: Efficacy and Perspectives

### 7.1. Current Evidence of Botox Injection in Treating Male LUTS/BPH

BPH is one of the main contributing diseases to LUTS in older men. However, not all men with LUTS are treated satisfactorily with BPH medication. Transurethral resection of the prostate is an established surgery for the rapid relief of LUTS in patients with BPH and bladder outlet obstruction (BOO). However, because of potential complications such as erectile dysfunction or urinary incontinence, patients might not accept this procedure. Therefore, minimally invasive procedures have been developed and tried in men with LUTS suggestive of BPH.

Several preliminary clinical trials have been reported. The injection of 200 U of Botox into the prostate has been shown to be effective with minimal side effects in patients with BPH and BOO who are poor surgical candidates [122]. Further clinical trials also revealed that Botox could relieve LUTS in patients with a small BPH of <30 mL [123]. Another study showed that an injection of 200 U of Botox into the prostate could improve LUTS and reduce the prostate volume by 50% in patients with large BPH, and the effect lasted for 12 months [15]. The transurethral injection method has been recommended as a preferable technique [124].

Several nonrandomized clinical studies have shown that Botox injection to the prostate can relieve LUTS in men with a small or large BPH. Intraprostatic Botox-A injection can reduce prostate volume, increase maximum flow rate and voided volume, and decrease PVR [125,126]. Patients with an International Prostate Symptom Score (IPSS) of ≤22, a Qmax ≤ 10 mL/min, and a prostate volume ≤ 56.5 mL had better treatment outcomes [127]. These results indicated that in patients with BPH, intraprostatic Botox injection is safe and effective for improving LUTS and quality of life. However, a randomized, double-blind, placebo-controlled clinical trial revealed an effect on LUTS/BPH symptoms, including IPSS, total prostate volume, transition zone volume, and Qmax, in both the Botox and placebo groups at week 12. Therefore, this study concluded that the therapeutic effects of Botox on BPH/LUTS are merely a placebo effect [128]. The results of a systematic review and meta-analysis of Botox injection for LUTS/BPH also showed no difference in the efficacy between Botox and a placebo. Thus, current evidence does not support the use of Botox injection for LUTS/BPH in real clinical practice [129].

### 7.2. Myth or Truth of Botox on BPH: Are We Treating Urethral Smooth Muscle or Prostatic Gland?

A previous study in patients with urethral sphincter pseudodyssynergia after cerebrovascular accidents or intracranial lesions reported that urethral sphincter Botox injection was effective and had no adverse effects [112], suggesting that Botox can relax the urethral smooth muscle or striated muscle and facilitate spontaneous voiding. Injecting Botox into the bladder neck and urethra has been shown to improve LUTS and increase Qmax in men with LUTS and a small prostate [130]. Botox prostatic injection has been demonstrated to be a promising treatment for patients with small prostates and symptomatic BPH. The mean prostate volume, symptom score, and quality-of-life index were significantly reduced after treatment. Because LUTS in men with a small BPH might relate more to urethral smooth muscle rather than to the prostate gland itself, the therapeutic effect of Botox on LUTS/BPH might relate to urethral dysfunction more than prostatic obstruction [131]. All of these pilot studies showed that LUTS does not result solely from BPH and obstruction. The functional inhibition of the voiding reflex or obstruction of the bladder outlet might play important roles in male LUTS, and Botox may eliminate these dysfunctions and improve LUTS. Despite the diverse results of clinical studies of Botox on LUTS/BPH, based on the current data, intraprostatic Botox still can be considered as a promising, safe, and minimally invasive procedure for patients with BPH who are not suitable for surgical intervention and who have an unsatisfactory response to standard drug therapy [132,133].

### 7.3. Perspectives of Botox Injections for Male LUTS/BPH

For men with symptomatic BPH, Botox injection into the prostate is a minimally invasive, safe, and effective procedure. The mechanisms of relief of LUTS might not depend completely on reducing prostate volume [123]. A previous study of Botox urethral-sphincter injection in patients with voiding dysfunction revealed that 61.1% of patients could benefit from this treatment [134]. In women with voiding dysfunction, Botox urethral injection can significantly improve LUTS without altering Qmax and voiding detrusor pressure [135]. These results indicate that the inhibitory effect on urethral smooth muscle or abnormal sensory function might play an important role in the therapeutic effects of Botox on voiding dysfunction and LUTS [123]. Furthermore, whether the dosage of Botox can affect therapeutic efficacy has not been well elucidated [136].

Based on the above evidence, there are several critical points that should be addressed in the future to clarify the therapeutic role of Botox on male LUTS/BPH: (1) the therapeutic effect of Botox injection to the bladder neck in the treatment of urodynamically proven bladder neck dysfunction in male LUTS; (2) the therapeutic effect of Botox prostatic injections between male patients with LUTS and different prostatic volumes and obstructive severity; (3) the therapeutic efficacy of Botox urethral injection in male patients with LUTS who have a small prostate volume and urodynamic BOO and non-BOO; and (4) the treatment outcomes of different doses of Botox (100 U or 200 U) and injecting sites (prostate gland or prostatic urethral smooth muscle) in male patients with LUTS who have a small prostatic volume.

## 8. Potential Clinical Applications of Botox on Recurrent UTI, Detrusor Underactivity, and AD

### 8.1. Will Botox Injection Reduce Episodes of UTI in Neurogenic LUTD?

Deficits and inflammation in the bladder urothelial barrier have been found to be increased in patients with chronic SCI, resulting in chronic inflammation and increased apoptosis and contributing to recurrent UTI in patients with NDO and DSD [137]. The expressions of the γEpithelial Na(+) channel and the acid-sensing ion channel 1 in the urothelium of patients with NDO have been found, which might have an impact on impaired mechanosensory function and low bladder compliance [138]. Because Botox can reduce detrusor pressure and episodes of involuntary detrusor contractions, intravesical Botox injections may decrease the incidence of symptomatic UTI in patients with NDO and low bladder compliance [139]. Women with recurrent UTI may have different voiding dysfunction due to DO or DV, resulting in damage to the integrity of the urothelial barrier and invasion by uropathogens [139]. Injecting Botox into the urethral sphincter may also decrease urethral resistance and voiding detrusor pressure, and patients with OAB and DV might experience fewer UTI episodes after Botox injections [140].

### 8.2. Risk of UTI after Botox Injection in Patients with OAB and IC/BPS

UTI is a major complication after intravesical Botox injections for NDO, OAB, and IC/BPS. Although UTI can be treated with antibiotics, the occurrence of this adverse event might prohibit patients from receiving this treatment after their first UTI experience following Botox injection. A recent study reported that the administration of a first Botox injection within 30 days of a UTI does not increase the risk of post-Botox UTI [141]. Patients with prior prolapse surgery or with recurrent UTI may have a higher risk of UTI after a Botox procedure [142]. Actually, Botox injection may improve bladder and bladder outlet functions, resulting in a reduction in UTI incidence [143]. In patients with NDO with 20 or 30 sites of Botox injections, the most common bacterium detected was *Escherichia coli*; however, the incidence of UTI was similar between groups [144].

### 8.3. What Are the Predictors for Successful Botox Treatment on DU?

Because Botox injection may decrease urethral resistance, it has been hypothesized that patients with DU or detrusor acontractility might benefit from urethral Botox injections. Chinese botulinum toxin A, Prosigne^®^, had been shown to be effective for treating an underactive bladder. After Botox injection, the Qmax increased, maximum urethral pressure decreased, and PVR decreased; however, the therapeutic effect seemed to last for only 3 months [145]. In a recently published article, female patients with DU showed improvement in voiding efficiency after urethral Botox injections, but patients with very low detrusor contractility, an absence of bladder sensation, and a tight bladder neck in a videourodynamic study showed less-favorable treatment outcomes [146].

Urethral sphincter Botox injections for voiding dysfunction were found to be effective in 60% of patients with DU, including 74.1% of patients with non-neurogenic DU and 48.5% of patients with neurogenic DU, yet the duration of the therapeutic effect was similar between patients with non-neurogenic and neurogenic DU [147]. The good treatment outcome was not related to age, gender, or videourodynamic subtypes. An open bladder neck during straining to voiding was the key factor in a successful result [147].

### 8.4. Perspectives of Botox Injections for Autonomic Dysreflexia (AD) in Patients with SCI and Neurogenic LUTD

Several issues are difficult to treat using medication or surgery. Because Botox has an anti-inflammatory mechanism of action, LUTDs related to chronic inflammation might be treated with Botox injection at the target organ or afferent nerves to achieve a satisfactory result [148]. In patients with chronic SCI, AD is a challenge for urological management, and Botox injection might reduce AD severity and improve the quality of life of these patients. There are some important questions to be resolved in future research: (1) Is AD an indication for Botox injection in patients with spinal cord lesion? (2) What is the optimal dose of Botox, and how frequently should patients be injected? (3) What is the therapeutic efficacy of the treatment of AD between injecting Botox into the detrusor and urethral sphincter? (4) Can Botox injection reduce the severity of AD after AE for patients with SCI who have a severely contracted bladder and AD?

Table 1 lists the clinical applications of Botox on lower urinary tract diseases or dysfunctions, and the dose, route, indications, adverse events after Botox injection or instillations. Although Botox has been launched for treatment of urological diseases for more than three decades, there should have more we can learn from the past researches and clinical trials. Through modification of dose adjustment, injection techniques, and the help of vehicles to carry Botox protein into the tissue, there should have more applications in the future to solve some urological diseases and dysfunctions that are not appropriately treated by the conventional medications.

**Table 1:** Current application of botulinum toxin A on lower-urinary-tract diseases or dysfunctions.

LUTD	Dose and Route	Indications	Adverse Events	References
**Overactive bladder**	100 U detrusor	Urinary incontinence Frequency urgency	Difficult urination Urine retention, UTI	[2,4,7,8,9,10,11,12,15,16,18,19,33,35,36,37,38]
**Overactive bladder**	200 U liposome encapsulated	Urinary incontinence Frequency urgency		[26,27]
**Neurogenic DO**	200 U–300 U	SCI, MS with UUI	Need CIC, UTI	[41,42,43,44,45,46,47,48,49,50,51,54,55,56,61,99]
**IC/BPS**	100 U detrusor	IC/BPS, ketamine cystitis	Difficult urination Urine retention, UTI	[70,71,72,74,76,77,78,79,80,81,82,83,84]
**IC/BPS**	100 U liposome encapsulated	IC/BPS	UTI	[29,85]
	200 U LESW	IC/BPS		[32]
**Pediatric NDO and OAB**	5 U/Kg detrusor	Non-neurogenic OAB, Urinary incontinence	UTI	[52,86,87,88,89,90,91,92,93,94,95,96,97,98,101]
**Neurogenic DSD**	100 U urethral sphincter	Dysuria, urine retention	Urinary incontinence, UTI, AD	[40,57,103,104,105,106,109,110,111,112,113,114,115]
**Neurogenic AD**	100 U bladder neck 200 U detrusor	Dysuria, AD	UTI	[148]
**Male LUTS/BPH**	200 U prostate	Dysuria, urine retention	UTI	[122,123,124,125,126,127,128,129,131,132,133,136]
**Bladder neck dysfunction**	100 U bladder neck	Dysuria,	Urinary incontinence	[130]
**Non-neurogenic voiding dysfunction**	100 U bladder neck, urethral sphincter	DV, DU, PRES	Urinary incontinence, UTI	[107,108,111,116,117,118,119,120,121,134,135,145,146,147]

## 9. Conclusions

Botox has been used in functional urology for more than 20 years. The licensed applications are limited to the treatment of OAB and NDO. However, because the pharmacologic mechanisms include inhibiting the release of neuropeptides, neuromodulation, anti-inflammatory effects, and antisense actions, Botox can be used in various LUTDs that are difficult to treat using conventional medications or surgical procedures. Advancing the clinical applications of Botox in LUTD necessitates further clinical and basic research to broaden the scope of its therapeutic effects.

## Abbreviations

AD	autonomic dysreflexia
AE	augmentation enterocystoplasty
BOO	bladder outlet obstruction
Botox	botulinum toxin A
BPH	benign prostatic hyperplasia
DO	detrusor overactivity
DSD	detrusor–sphincter dyssynergia
DV	dysfunctional voiding
IC/BPS	interstitial cystitis/bladder pain syndrome
IPSS	International Prostate Symptom Score
LESW	lower-energy shock wave
LUTD	lower-urinary-tract dysfunction
LUTS	lower-urinary-tract symptoms
MS	multiple sclerosis
NDO	neurogenic detrusor overactivity
OAB	overactive bladder
Pdet	detrusor pressure
PRES	poorly relaxed external sphincter
PTNS	percutaneous tibial nerve stimulation
PVR	post-void residual
Qmax	maximum flow rate
SCI	spinal-cord injury
SNM	sacral-nerve neuromodulation
UTI	urinary-tract infection
UUI	urgency urinary incontinence
